# Dialectical behavior therapy skills use and emotion dysregulation in personality disorders and psychopathy: a community self-report study

**DOI:** 10.1186/s40479-016-0041-5

**Published:** 2016-07-20

**Authors:** Andrada D. Neacsiu, Mathew A. Tkachuck

**Affiliations:** Clinical-Disaster Research Center, University of Mississippi, Kinard Hall, Suite 203, University, MS 38677-1848 USA; Duke University Medical Center, 2213 Elba St., Rm 123, Durham, NC 27710 USA

**Keywords:** Emotion dysregulation, Transdiagnostic, Skills use, Personality disorders, Psychopathy

## Abstract

**Background:**

Emotion dysregulation is a critical transdiagnostic mental health problem that needs to be further examined in personality disorders (PDs). The current study examined dialectical behavior therapy (DBT) skills use, emotion dysregulation, and dysfunctional coping among adults who endorsed symptoms of cluster B PDs and psychopathy. We hypothesized that skills taught in DBT and emotion dysregulation are useful for adults with PDs other than borderline personality disorder (BPD).

**Methods:**

Using a self-report questionnaire, we examined these constructs in three groups of community adults: those who reported symptoms consistent with borderline personality disorder (BPD; *N =* 29), those who reported symptoms consistent with any other cluster B PD (*N* = 22), and those with no reported cluster B PD symptoms (*N* = 77) as measured by the Personality Diagnostic Questionnaire-4 + .

**Results:**

Both PD groups reported higher emotion dysregulation and dysfunctional coping when compared to the no PD group. Only the BPD group had significantly lower DBT skills use. DBT skills use was found to be a significant predictor of cluster B psychopathology but only before accounting for emotion dysregulation. When added to the regression model, emotion dysregulation was found to be a significant predictor of cluster B psychopathology but DBT skills use no longer had a significant effect. Across all groups, DBT skills use deficits and maladaptive coping, but not emotion dysregulation, predicted different facets of psychopathy.

**Conclusion:**

Emotion dysregulation and use of maladaptive coping are problems in cluster B PDs, outside of BPD, but not in psychopathy. Inability to use DBT skills may be unique to BPD. Because this study relied exclusively on self-report, this data is preliminary and warrants further investigation.

## Background

### Difficulties with emotion regulation as a transdiagnostic mental health problem

With the advent of the National Institute of Mental Health (NIMH) Research Domain Criteria Project (RDoC; [80]) in 2008, there has been a shift in understanding and treating mental disorders via identifying and addressing underlying core deficits that are transdiagnostic. The primary goal of the RDoC is to establish a fundamental understanding of psychopathology by applying methodologies from multiple fields of study concurrently (e.g., genetics, neuroscience, and behavioral science), and creating a dimensional, yet adaptable, system of classification that cuts across disorders [80]. Several core problems that cut across diagnoses have been identified (e.g., [37, 50]) and several treatments have emerged that address transdiagnostic problems such as the Unified Protocol [23] or Dialectical Behavior Therapy Skills Training (DBT-ST; [56]).

Despite not being included in the RDoC framework, emotion dysregulation is one of the most widely supported transdiagnostic mechanisms of disorders. One influential contemporary model defines emotion dysregulation as a multidimensional set of difficulties with: (1) emotional awareness, (2) clarity, (3) acceptance, (4) impulsivity, (5) achieving goals, and (6) use of emotion regulation strategies when upset [31]. Such difficulties with regulating emotions occur across psychopathology (e.g., [1, 50]), mediate [27, 83] and moderate [2, 16] development and course of mental illness. In addition, problematic behaviors such as suicidality [18], self-injury [30], substance use [84], binging/ purging [50], and impulsive action [85] serve emotion regulation functions. Therefore, emotion dysregulation is a critical transdiagnostic mental health problem.

### Personality disorders include problems with managing emotions

Most of the theoretical and empirical work on transdiagnostic mechanisms of disorder and treatment has covered affective disorders (mood, anxiety) and borderline personality disorder (BPD). Nevertheless, other personality disorders (PDs) are likely to have underlying transdiagnostic processes. A PD is a pervasive pattern of behaviors, that cause a significant amount of psychological distress and impairment in functioning across a variety of contexts, and that is stable over the life course [4]. The National Epidemiological Survey on Alcohol and Related Conditions [86] found prevalence rates of PDs, ranging from 9.1-15.0 % using the *Diagnostic and Statistical Manual of Mental Disorders, 4*^*th*^*Edition Text Revision (DSM-IV-TR)* criteria [3]. The definition of PDs slightly changed in the recently released *DSM-5* [4]; nevertheless, these changes are not likely to affect the prevalence of PDs.

PDs are complex and severe disorders. Researchers have documented cognitive deficiencies [40], physical impairments and disability [68], frequent comorbidity [36, 78], and frequent help seeking behaviors [43] in adults with PDs. Therefore, adults who meet criteria for these disorders are typically very difficult to treat and utilize a significant amount of resources [69]. Therefore, PDs are a major public health problem that cause significant burden on the United States economy.

Several studies point towards difficulties with managing emotions as one core characteristic of PDs. Trull et al. [86] found that adults meeting criteria for PDs had more emotional problems that interfered with their overall health, had higher amounts of perceived stress, and reported having significantly less social support. Hartford et al. [36] also found significant correlations between meeting criteria for a PD and several affective disorders such as social phobia, major depression, or generalized anxiety disorder. Furthermore, high rates of suicidal behaviors in adults who meet criteria for PDs [86] also suggest possible problems managing emotions [49]. Therefore, examining emotion dysregulation across personality disorders is warranted.

### Investigating emotion dysregulation in narcissistic, histrionic, and antisocial PDs

Of all PDs, there has been extensive research connecting BPD to emotion dysregulation. Theoretical and empirically established difficulties with emotional reactivity, sensitivity, and slow return to baseline have led researchers to name BPD an emotion dysregulation disorder [29, 52]. Adults diagnosed with BPD report high levels of negative emotion, increased sensitivity to emotional stimuli [72], decreased emotional awareness [51], more disproportionate use of emotion suppression [8], and difficulty not engaging in impulsive action when emotionally aroused [45] relative to healthy controls. Use of maladaptive behaviors commonly seen in those diagnosed with BPD, including suicide attempts, suicide threats, self-inflicted injuries, impulsive behaviors, dissociation, are shown to serve an emotion regulation function [11]. Therefore, emotion dysregulation is a core component of BPD.

Because of descriptive similarities, BPD, antisocial PD (ASPD), narcissistic PD (NPD), and histrionic PD (HPD) were grouped together in the *DSM-IV-TR* under the label of “Cluster B Personality Disorders”. This group of disorders was characterized more broadly as including emotional instability, impulsivity, and frequent engagement in maladaptive behaviors [3]. An epidemiological study conducted in 2010 found that, among over 40,000 US adults, 5.5 % met criteria for at least one of the cluster B PDs: 3.8 % for ASPD, 2.7 % for BPD, 1 % for NPD, and 0.3 % for HPD [86].

A review of the literature suggests that adults diagnosed with any of the cluster B PDs may have difficulties with emotion management. First, BPD has been recognized as a disorder of emotion dysregulation (e.g., [52]) and the high comorbidity between BPD and HPD, NPD, and ASPD (e.g., see [5, 13]) may indicate that pervasive emotion dysregulation is also present in these other PDs. Second, a diagnosis of NPD has been associated with emotional distress, anxiety, depression, and problematic behaviors that frequently serve emotion regulation functions (e.g., suicidal behavior, interpersonal conflict, increased substance use; [10, 55]; [74, 75, 90]). Third, research investigating characteristics of ASPD have also found significant associations with emotion regulation deficits as evidenced by aggressive, violent and criminal behaviors, substance abuse, depression, and self-harming behaviors [22, 36, 39, 73]. Finally, in regards to HPD, while there is a paucity of research investigating the etiology of HPD clinical problems, both historical conceptualizations (see [66]) and more recent studies [5, 79] illustrate the presence of emotion regulation-related problems with adults diagnosed with HPD, such as emotional instability, catastrophizing (i.e., fear-related cognitions that often lead to distress and maladaptive behaviors), attention seeking behavior (i.e., driven by feeling distressed when not the center of attention), and difficulties in self-regulating. Taken together these findings suggest that adults diagnosed with HPD, NPD or ASPD may have emotion dysregulation.

### Investigating emotion dysregulation in psychopathy

While not considered a formal diagnosis in the *DSM*, psychopathy is a complex PD associated with interpersonal and affective deficits as well as socially deviant and destructive behaviors [33]. One model of psychopathy with empirical support highlights four types of problems commonly encountered in psychopathy: (1) interpersonal (e.g., manipulative, grandiose sense of self-worth), (2) affective (e.g., lack of guilt, empathy, and remorse), (3) erratic lifestyle (e.g., impulsive, irresponsibility), and (4) antisocial acts (e.g., past and current criminal behaviors; [35]). The destructive behavior (e.g., criminal and violent offences) associated with psychopathy leads to high societal and economical cost, especially when considering the high recidivism rates [47, 70]. Although psychopathy is an entity of its own, the term is often used interchangeably with the ASPD diagnosis due to shared characteristics such as the extensive “disregard for, and violation of, the rights of others” ([3], p. 701).

Psychopathy is also a candidate PD that may be related to emotion dysregulation. Several studies indicate that there are significant associations between the cluster B PDs and psychopathy ([13, 41]; [71]). More specifically, Casey et al. [15] found that psychopathic individuals higher on the callous/unemotional facets displayed deficits in experiencing negative emotions. This is important given that emotional awareness and clarity, and the willingness to experience emotions (acceptance) are key components of the emotion regulation model [31]. In addition, higher levels of psychopathic traits have been associated with the use of maladaptive emotion regulation strategies and, for those who reported more grandiose and manipulative behaviors, increased emotional problems [38]. Furthermore, behaviors that may indicate emotion dysregulation (i.e., interpersonal violence, substance use, suicidal behavior) are also common in adults who score high on psychopathy [21, 48, 61]. However, despite evidence supporting a potential relationship between psychopathy and emotion dysregulation, historically, psychopathy has been understood as a PD that is deficient in emotion altogether [17, 34]. Therefore, more research investigating the potential relationship between emotion dysregulation and characteristics of psychopathy is needed.

### Lack of behavioral skills to manage emotions May be responsible for emotion dysregulation

Based on the conceptualization of BPD as a disorder of emotion dysregulation, Linehan [52] also hypothesized that such difficulties emerge because of lacking psychological skills to manage emotions effectively across contexts. Empirical examinations of this hypothesis support that use of the skills taught in Dialectical Behavior Therapy (DBT; [52]), an evidence-based treatment for BPD, may be a mechanism of change for emotion dysregulation in BPD [59] and in emotionally dysregulated adults diagnosed with depression or anxiety [56]. The four skills taught in DBT include emotion regulation, interpersonal effectiveness, distress tolerance, and mindfulness [52].

Similar to the lack of research linking emotion regulation deficits with cluster B PDs and psychopathy, few studies examined DBT skills use in NPD, HPD, ASPD, or in psychopathy. Emerging theoretical articles describe how DBT skills can be applicable to PDs, especially to ASPD [81], or to psychopathic offenders [28]. These conceptualizations are promising for furthering transdiagnostic research on using DBT skills to address some of the core problems that are prevalent across disorders, such as those mentioned previously, but have offered no empirical evidence thus far about DBT skills deficits in these clinical populations. Therefore, more research is needed to examine the relationship between these disorders, emotion dysregulation, and deficits in DBT skills.

### Current study

The current study aims to further examine DBT skills deficits and emotion regulation difficulties among the cluster B PDs and facets of psychopathy. Community adults completed an online questionnaire including measures of emotion dysregulation, DBT skills use, psychopathy, and cluster B PDs. We separated our sample into three groups: a) those who self-reported symptoms consistent with a BPD diagnosis (BPD group; i.e., either a sole diagnosis of BPD or BPD comorbid with any other of the cluster B PDs), b) those who self-reported symptoms consistent with a ASPD, NPD, or HPD diagnosis (otherPD group; i.e., including comorbidity between these PDs but excluding comorbidity with BPD), and c) those who did not report enough symptoms consistent with any cluster B PD (noPD group). We hypothesized that the otherPD and the BPD groups would report significantly higher emotion dysregulation and lower use of DBT skills than the noPD group. We did not expect any significant differences between the otherPD and the BPD groups on these measures.

In addition, the current study aimed to investigate the relationship between emotion dysregulation, DBT skills use, and subscales of psychopathy. While, conventionally, measures of psychopathy are analyzed as predictors of outcomes, consistent with theories of personality that emphasize the importance of context [6, 54], we assumed that psychopathy dimensions are not immutable and may reflect behavioral patterns commonly seen in cluster B personality disorders (e.g., difficulties with emotion regulation may play a role in endorsement of criminal behaviors). Therefore, we were interested in examining whether difficulties with emotion regulation and lack of DBT-related skillful behavior explain variance in callousness, interpersonal manipulation, criminal tendencies, and erratic life styles.

We hypothesized that, a) emotion dysregulation would be a significant predictor of increased cluster B psychopathology, b) DBT skills use would be a significant predictor of reduced emotion dysregulation, and c) while DBT skills use would be a significant predictor of reduced cluster B psychopathology, that relationship would be significantly accounted for by its reduction in emotion dysregulation. In addition, we expected that DBT skills use and emotion dysregulation will be significant predictors of all the facets of psychopathy.

## Methods

### Participants

Participants were residents of the United States, who reported being older than 18 years of age. All participant data was de-identified.

### Procedure

Participants were recruited via flyers posted in a large Northwestern city, on online bulletin boards and Listervs of departments within the University of Washington. We also advertised in local newspapers and on Craigslist. The recruitment materials advertised the study including the possibility to participate in a raffle for a $50 gift card. Interested participants were provided with a link to an online questionnaire that included all the study measures and an information statement detailing the informed consent process. All procedures were approved by the University of Washington Institutional Review Board. Data was collected between 08/2011 and 09/2012.

### Measures

#### Personality Diagnostic Questionnaire-4 (PDQ-4+)

The PDQ-4+ [42] is a self-report measure that is widely used to assess the DSM-IV criteria for PDs. It consists of 99 True-False items including a scale for each PD and two validity scales: the Too Good (TG) scale, designed to identify under-reporting, and the Suspect Questionnaire (SQ) scale, designed to identify lying or responding randomly. In the present study, only the cluster B PD scales and the two validity scales were administered. The validity scales were employed as recommended in the PDQ manual [42].

Test-retest reliability for the PDQ-4+ cluster B PD scales, using Pearson *r* correlations, ranged from .47 to .87 over a nine-day period assessed across three time points [60]. Some evidence of convergent validity was found with the Structured Clinical Interview for DSM-IV Axis II Personality Disorders [25] with correlations ranging from .29 to .42 in relation to the cluster B PDs [26]. Although current recommendations argue for inclusion of clinical interviews in order to diagnose PDs [26], we decided to include the PDQ-4+ because the measure is widely used, it includes validity scales that provide information about the integrity of the self-report data, and there is no other self-report measure with stronger psychometric properties for cluster B PDs.

#### Self-Report Psychopathy Scale-Short Form (SRP-SF)

The SRP-SF [64] is a self-report analogue of the Psychopathy Checklist-Revised (PCL-R; [34]), where participants are asked to rate 29 items on a 5-point Likert-type scale. The measure includes four subscales: callous affect (CA), interpersonal manipulation (IPM), erratic lifestyle (ELS), and criminal tendencies (CT). The SRP has evidence for good construct validity as indicated by significant correlations with relevant self-report personality [88] and psychopathy measures [64]. In addition, Carre et al. [14] reported acceptable internal consistencies for all four facets (*α*s > .74). In the current study, internal consistencies for all four facets were in the acceptable range or above except for the CT subscale (i.e., α_CT_ = .66, α_CA_ = .77, α_IPM_ = .78, α_ELS_ = .82).

#### Difficulties in Emotion Regulation Scale (DERS)

The DERS [31] contains six dimensions of emotion dysregulation, including: (a) lack of awareness (*awareness)* and (b) clarity of emotional responses (*clarity)*, (c) non-acceptance of emotional responses (*nonacceptance)*, (d) limited access to emotion regulation strategies perceived as effective (*strategies)*, (e) difficulties controlling impulses (*impulse)* and (f) engaging in goal-directed behaviors when experiencing negative emotions (*goal-directed)*. The DERS total score has high internal consistency (*α* = .93) and all of the DERS subscales have adequate internal consistency (*α*s > .80; [31]). The DERS also has evidence for good test-retest reliability over periods of 4-8 weeks (*ρ* = .88, *p* < .01) and for adequate construct and predictive validity [31]. In this study we found acceptable internal consistencies for each subscale: .82 for lack of awareness, .87 for lack of clarity, .83 for non-acceptance, .85 for limited strategies, .90 for difficulties controlling impulses, .80 for difficulties goals, and .96 for the total DERS score.

#### Dialectical Behavior Therapy-Revised Ways of Coping Checklist (DBT-WCCL)

The DBT-WCCL [58], is a 59-item self-report of the frequency of adaptive and maladaptive skills used to manage difficult situations over the past month with two subscales measuring DBT skills use and dysfunctional coping. Items assess DBT skills use independent of the DBT language and therefore, the measure is applicable to those who have no prior exposure to treatment. Examples of items include, “Just took things one step at a time”, “Focused on the good things in my life”, and “Came up with a couple of different solutions to my problems”. In several BPD samples, the DBT skills use subscale (DSS) had excellent internal consistency (Cronbach’s *α*s = .92 – .96; *n* = 316). Test-retest reliability at 4 months for 119 men and women diagnosed with BPD treated without access to DBT skills training was excellent, (*r =* .71, p < .001).

Outside of BPD samples, the DBT-WCCL also had excellent internal consistency (Cronbach’s *α*s = .94; *n* = 228) in a heterogeneous psychiatric sample consisting of participants endorsing an array of mood, anxiety, and personality disorders [82]. Stein et al. [82] also reported the DBT-WCCL DSS scale having good convergent validity with the Cognitive Behavior Therapy Skills Questionnaire ([44]; *r =* .62, p < .01) and discriminant validity with the Multidimensional Outcome Expectations for Exercise Scale ([89]; *r =* .18, p = .01). These findings suggest strong psychometric properties and validity of the DBT-WCCL across diagnostic samples. In the current study internal consistency for both subscales was high (i.e., αs > .90).

## Results

A total of 211 participants residing in the United States took part in this study. Using the information collected on the PDQ-4+ we first assessed the validity of our sample’s responses, in accordance with the PDQ+ scoring instructions, and excluded 83 participants for underreporting, lying, or answering randomly. Of the included 128 respondents (*M*_age_ = 31.18, *SD*_age_ = 12.04), the majority were Caucasian (74.2 %), women (75.0 %) who had graduated from college (57.0 %), were single, separated, or divorced (78.9 %) and, in the last year, either earned more than $30,000 (33.6 %) or less than $5,000 (21.9 %). Using the PDQ-4+ subscales, we identified 29 participants who reported symptoms consistent with BPD (i.e., > 4 BPD symptoms; the BPD group); 22 that reported symptoms consistent with either NPD (i.e., > 4 NPD symptoms), ASPD (i.e., > 3 symptoms of conduct disorder and > 3 ASPD symptoms since age 15), or HPD (i.e., > 4 HPD symptoms; the otherPD group); and 77 who did not report symptoms consistent with any of the cluster B PDs (noPD group). See Table 1 for demographic descriptors by group.

**Table 1:** Demographics by group

	No PD	BPD	OtherPD
	(*n* = 77)	(*n* = 29)	(*n* = 22)
Mean Age (SD)	31.91 (12.82)	28.07 (11.23)	32.73 (9.73)
Female	74.0 %	79.3 %	72.7 %
Caucasian	77.9 %	69.0 %	68.2 %
African American	2.6 %	3.4 %	4.5 %
Asian/Asian American	6.5 %	20.6 %	22.6 %
Other ethnic background	11.7 %	6.9 %	4.5 %
Hispanic or Latino	5.2 %	3.4 %	0.0 %
High school graduate/GED	2.6 %	13.7 %	9.0 %
Some college	22.1 %	51.7 %	40.9 %
College graduate	71.4 %	31.0 %	40.9 %
Single, separated, or divorced	75.3 %	89.6 %	77.3 %
Earn < $5,000/year	23.4 %	20.7 %	18.2 %
Earn ≥ $30,000/year	46.7 %	10.3 %	18.1 %

*Note.* No PD = those who did not meet criteria for any cluster B PD; BPD = those who reported high levels of BPD traits, either high levels of BPD traits exclusively or BPD traits comorbid with high levels of any other of the cluster B PD traits; Other PD = those who reported high levels of ASPD, NPD, or HPD traits including comorbidity between these PDs but excluding comorbidity with BPD

All variables included in the analyses were normally distributed according to the Shapiro-Wilk normality analyses (*W* > .90) except for age (*W* = .85; *p* < .001; skewness =1.45, *S.E. =* .22; kurtosis = 1.94, *S.E.* = .43) and the CT subscale of the SRP (*W* = .76; *p* < .001; skewness = 1.59, *S.E.* = .22; kurtosis = 2.03, *S.E. =* .43). Age, was transformed using a square root function to achieve normality. For the CT subscale of the SRP common transformations did not lead to a normal distribution. Therefore, we transformed the CT score into a binary variable: no criminal tendencies vs. any criminal tendencies. No significant difference emerged between groups on any of the demographic variables and therefore, they were not included as covariates in any subsequent analyses (*p*s > .05). See Table 2 for correlations and means of the primary variables.

**Table 2:** Correlation analyses for primary outcomes and facets of psychopathy

			Bivariate Correlations						
	*M*	*SD*	1	2	3	4	5	6	7
DERS total	83.76	25.80	_						
DBT Skills Use	1.92	.40	-.36**	_					
Dysfunctional Coping	1.61	.55	.69**	.03	_				
*SRP Subscales:*									
Interpersonal manipulation	1.96	.69	.26*	-.27*	.26*	_			
Callous affect	1.91	.68	.35*	-.24*	.43*	.63**	_		
Erratic lifestyle	2.18	.72	.39**	-.14	.44**	.52**	.70**	_	
Criminal tendencies	1.29	.39	.27*	-.13	.29*	.51**	.57**	.56**	_

*Note. DERS* Difficulties in Emotion Regulation Scale; *SRP* Self-Report Psychopathy; *DBT* Dialectical Behavior Therapy; * *p* < .01. ** *p* < .001

A multivariate analysis of variance was conducted using emotion dysregulation, DBT skills use, and dysfunctional coping as outcomes, and group (noPD, BPD, otherPD) as the independent variable. A Bonferroni correction for multiple comparisons (*p =* .05/3 = .016) was employed. Box’s test (*M* = 9.89; *F*[12, 17197.79] = .78, *p* = .67) indicated that the covariance matrices of the dependent variables were not significantly different across levels of the independent variable; therefore, no additional correction was needed. Multivariate analyses indicated a statistically significant main effect of group, *F*(6, 242) = 16.33, *p* < .001; Wilk's λ = 0.51, partial ε^2^ = .29. Between subjects analyses indicated a significant main effect for emotion dysregulation (*F*[2, 123] = 54.94, *p* < .001; partial ε^2^ = .47), dysfunctional coping (*F*[2, 123] = 25.63, *p* < .001; partial ε^2^ = .29), and for DBT skills use (*F*[2,123] = 6.04, *p* = .003; partial ε^2^ = .09).

Post-hoc analyses comparing the otherPD, BPD, and noPD groups found significant differences on all variables of interest. The BPD group reported significantly higher difficulties with emotion regulation than the otherPD group who, in turn, reported significantly higher emotion dysregulation than the noPD group (*p* < .001). Only the BPD group reported significantly lower use of DBT skills than the noPD group (*p* = .001). Both the otherPD and BPD groups, reported comparable and significantly higher dysfunctional coping than the noPD group (*p*s < .016).

Because we found a significant main effect of emotion dysregulation, we conducted an exploratory analysis examining between group differences in the different subscales. We examined all analyses with the same corrected *p*-value of .016. Box’s test (*M* = 93.05; *F*[42, 11740.93] = 1.99, *p* < .001) indicated that the covariance matrices of the dependent variables were significantly different across levels of the independent variable; therefore, Pillai’s Trace was reported given the more robust nature of the statistic [62]. Multivariate analyses indicated a statistically significant main effect of group, *F* (12, 238) = 8.437, *p* < .001; Pillai’s Trace V = .60, partial ε^2^ = .30. Between subjects analyses indicated a significant main effect for *nonacceptance* (*F*[2, 123] = 26.29, *p* < .001; partial ε^2^ = .30), *awareness* (*F*[2, 123] = 5.16, *p* = .007; partial ε^2^ = .08), *clarity* (*F*[2, 123] = 22.34, *p* < .001; partial ε^2^ = .27), *goal-directed* (*F*[2, 123] = 12.97, *p* < .001; partial ε^2^ = .17), *impulse* (*F*[2, 123] = 51.20, *p* < .001; partial ε^2^ = .45), and *strategies* (*F*[2, 123] = 62.31, *p* < .001; partial ε^2^ = .50).

Post-hoc analyses comparing the otherPD, BPD, and noPD groups found significant differences on all of the DERS subscales except on the *awareness* subscale where all group scores did not significantly differ (*p*s > .016)*.* Both BPD and otherPD groups had similar but significantly more difficulties with emotional *acceptance* (*p* < .001 & *p* = .006 respectively) and emotional *clarity* (*p* = .001 & *p* < .001 respectively) than the noPD group. Only the BPD group expressed significantly more difficulties with *goal directed behavior* (*p* < .001) and *impulse control* when upset (*p* < .001), when compared to the noPD group. With regards to use of *strategies* to manage emotions, those with BPD reported a significantly higher impairment than those with otherPD (*p* < .001), who reported significantly higher impairment when compared to the noPD group (*p* = .001). See Table 3 for means and standard deviations for all primary outcomes, facets of emotion regulation and psychopathy.

**Table 3:** Means (SDs) for primary outcomes, facets of emotion dysregulation, and psychopathy scores

Variable	No PD	BPD	Other PD
	(*N* = 77)	(*N* = 29)	(*N* = 22)
DERS total	70.69 (16.84)	113.48 (21.69)	90.67 (21.68)
*DERS Subscale (Problems with):*			
Goal directed behavior	12.92 (5.04)	18.14 (4.36)	14.71 (3.74)
Use of strategies	14.28 (5.02)	27.86 (6.14)	19.24 (6.85)
Emotional awareness	13.40 (4.57)	15.82 (5.03)	16.48 (4.40)
Emotional clarity	9.41 (2.89)	14.43 (4.74)	12.62 (4.16)
Emotional acceptance	11.45 (4.65)	19.34 (5.90)	15.48 (5.87)
Impulsive behaviors	9.09 (3.18)	17.86 (5.42)	11.86 (4.38)
DBT Skills Use	2.01 (.36)	1.73 (.35)	1.85 (.49)
Dysfunctional Coping	1.39 (.45)	2.11 (.44)	1.73 (.55)
*SRP Subscales:*			
Interpersonal manipulation	1.84 (.60)	2.17 (.75)	2.11 (.86)
Callous affect	1.70 (.59)	2.29 (.66)	2.16 (.70)
Erratic lifestyle	1.94 (.60)	2.70 (.68)	2.36 (.78)
Criminal tendencies	1.18 (.28)	1.45 (.46)	1.47 (.52)

*Note.* No PD = did not meet criteria for any cluster B PD; BPD = reported symptoms consistent with a BPD diagnosis; Other PD = adults who reported symptoms consistent with an ASPD, NPD, or HPD diagnosis including adults who reported symptoms consistent with comorbidity between these PDs (excluding BPD); DERS = Difficulties in Emotion Regulation Scale; SRP = Self-Report Psychopathy; DBT = Dialectical Behavior Therapy

The relationships between DBT skills use, emotions dysregulation, and cluster B psychopathology were analyzed using two simple linear regressions and one hierarchical regression. Prior to the analyses, multicollinearity statistics were computed and results indicated that multicollinearity between the independent variables, DBT skills use and emotion regulation, were not a concern (*Tolerance* = .87, *VIF* = 1.15). Two participants were excluded from the subsequent analyses due to missing data, leaving a total of 126 participants. First, when controlling for DBT skills use, with emotion dysregulation entered as the independent variable and cluster B psychopathology as the dependent variable, results indicated that emotion dysregulation significantly predicted cluster B psychopathology (*β =* .13; *t*[123] = 8.59, *p* < .001). Second, with DBT skills use entered as the independent variable and emotion dysregulation as the dependent variable, DBT skills deficits were found to be a significant predictor of emotion dysregulation (*β =* -23.30; *t*[124] = -4.29, *p* < .001). A two-stage hierarchical linear regression was used to assess the relationship between DBT skills use and cluster B psychopathology while including emotion regulation in the model. DBT skills use was entered into the first block and emotion dysregulation was entered into the second block. Results suggest that before accounting for emotion dysregualtion, DBT skills use was a significant predictor of cluster B psychopathology (*β =* 2.51; *t*[124] = -2.15, *p* < .001). However, when emotion dysregulation was added to the model, DBT skills use no longer had a significant effect on cluster B psychopathology (*β =* .56; *t*[123] = .57, *p* = .573) while emotion regulation did.

To assess the relationship between DBT skills use, emotion dysregulation, dysfunctional coping, and psychopathy, we conducted three linear regression analyses with the CA, IPM, and ELS SRP subscales as dependent variables and one logistic regression analysis using the CT SRP subscale as the dependent variable. Because we tested three predictor variables for each of the four regression models, we used a Bonferroni correction of .05/12 and therefore examined significance at the .004 level in these analyses. For each linear regression model, DBT skills use, emotion dysregulation, and dysfunctional coping were entered simultaneously in step one. These analyses showed that: (a) DBT skills use and dysfunctional coping were significant predictors of CA with the full model, according to the *adjusted R*^***2***^*,* explaining 24.0 % of the variance in CA characteristics; (b) only DBT skills use was a significant predictor of IPM with the full model explaining 13.8 % of the variance in IPM characteristics; and (c) only dysfunctional coping was a predictor of ELS with the full model explaining 20.2 % of the variance in ELS characteristics (Table 4). The logistic regression showed that neither DBT skills use (*p* = .029) nor dysfunctional coping (*p* = .066) significantly predicted the likelihood of endorsing CT characteristics (Table 4). There were no significant effects found between emotion dysregulation and the four facets of psychopathy.

**Table 4:** Results from regression analyses using different types of predictors

Regression 1: Predictors of IPM	*F(3, 122)* = 7.67, *p* < .001, *R* ^*2*^ = .16		
	*β*	*t(122)*	*p*
Emotion Dysregulation	-.002	-.61	.544
DBT Skills Use	-.55	-3.22	.002
Dysfunctional Coping	.432	2.75	.007
Regression 2: Predictors of CA	*F(3,122)* = 14.26, *p* < .001, *R* ^*2*^ = .26		
	*β*	*t(122)*	*p*
Emotion Dysregulation	-.003	-.91	.363
DBT Skills Use	-.51	-3.27	.001
Dysfunctional Coping	.64	4.46	< .001
Regression 3: Predictors of ELS	*F(3,122)* = 11.54, *p* < .001, *R* ^*2*^ = .22		
	*β*	*t (71)*	*p*
Emotion Dysregulation	.002	.50	.618
DBT Skills Use	-.24	-1.39	.167
Dysfunctional Coping	.53	3.37	.001
Regression 4: Predictors of CT	*χ* ^*2*^ (3, *N* = 128) = 12.89, *p* = .005		
	*B(SE)*	*Wald*	*Exp(B)*
Emotion Dysregulation	-.001(.01)	.005	1.00
DBT Skills Use	-1.27(.58)	4.79	.28
Dysfunctional Coping	.97(1.42)	3.38	2.64

*Note: IPM* interpersonal manipulation, *CA* callous affect, *ELS* erratic lifestyle, *CT* criminal tendencies, *DBT* dialectical behavior therapy, significant *p*-values were adjusted for multiple comparisons: *p* < .004 (.05/12)

## Discussion

The present study is an examination of emotion regulation difficulties, DBT skills use deficits, and dysfunctional coping in cluster B personality disorders and facets of psychopathy. Compared to participants who did not meet criteria for any PDs, those who self-reported symptoms consistent with BPD or any other cluster B PD (HPD, NPD, ASPD) diagnosis on the PDQ-4+ reported significantly more difficulties with emotion regulation and higher levels of dysfunctional coping. Additionally, participants who self-reported symptoms consistent with BPD also reported significantly fewer DBT skills when compared to the no PD, NPD, ASPD, and HPD groups. Dysfunctional coping significantly predicted the callous affect and erratic lifestyle, but not criminal tendencies and the interpersonal manipulation facets of psychopathy; low use of DBT skills was a predictor of interpersonal manipulation and callous affect. Lastly, emotion dysregulation was not a significant predictor of psychopathy.

These results partially confirmed our hypotheses. Although those who endorsed BPD problems reported the highest emotion dysregulation, as predicted, those who reported symptoms consistent with NPD, ASPD, and HPD also evidenced difficulties with emotion regulation and engagement in dysfunctional coping. This finding is novel for HPD; nevertheless, it is consistent with existing literature that links NPD and ASPD to emotion dysregulation problems (e.g., [65, 91]) and problematic coping (e.g., [9]). Our hypothesis that the lack of DBT skills use will be seen in all cluster B PDs was not supported.

A closer examination of group differences on the different facets of emotion dysregulation highlights similarities and differences between the cluster B PDs. Except for problems with awareness, BPD participants reported significantly higher difficulties in all aspects of emotion regulation when compared to controls. In contrast, those in the NPD, ASPD, and HPD groups only showed impairments in emotional acceptance, clarity (similar to BPD), and use of emotion regulation strategies (better than BPD) when compared with controls. There was no evidence in our sample of impairments in goal directed behaviors and impulsivity when upset in NPD, HPD, and ASPD, although these difficulties were present in BPD, a finding consistent with the literature [12, 76]. Taken together, these findings add to the body of literature suggesting that emotion dysregulation has transdiagnostic relevance [56, 57] and should be considered in treating PD pathology beyond BPD. In particular, interventions aimed at increasing emotional acceptance and clarity that have been developed for BPD should have direct relevance to the other cluster B PDs. Similarly, although those in the NPD, HPD, and ASPD groups reported fewer impairments in identifying strategies to use when distressed when compared to the BPD group, they endorsed difficulties in this area above and beyond controls, suggesting that interventions that teach emotion regulation skills and encourage their use when distressed are also warranted for this group.

Dimensional differences of emotion regulation between PDs are not unique in the literature. For example, participants diagnosed with BPD, with and without comorbid avoidant PD (AVPD), reported similar but increased difficulties with engaging in goal directed behavior, having emotional clarity and inhibiting impulses when distressed when compared to participants with neither BPD or AVPD [32]. Furthermore, participants with comorbid BPD and AVPD reported significantly more difficulties accessing emotion regulation strategies than those with only AVPD, who in turn reported more than those with neither PD [32]. Our results add to this growing support for the transdiagnostic nature of emotion regulation and emphasize the importance of understanding the unique patterns in which different problems with emotion regulation can be seen in different psychiatric disorders.

Interestingly, reported use of DBT skills was significantly lower in the BPD group when compared to the other groups, but similar between those who endorsed other cluster B PDs and those in the no PD group. Nevertheless, those in the BPD, NPD, HPD, and ASPD groups endorsed similar levels of dysfunctional coping that were significantly higher than those reported by control participants. These findings may suggest that although excess of problematic behavior is common in cluster B PDs, deficits in DBT-related skillful behavior may be unique to BPD (i.e., BPD individuals lack the life skills that are taught in DBT). It is also possible that the measure for use of DBT skills may not be best fitted to capture DBT skills deficits in participants that reported symptoms consistent with other cluster B PDs and that it may be uniquely tailored for BPD. Some past evidence for NPD and ASPD suggested a different clinical mechanism in these disorders when compared to BPD [77], which may support this hypothesis.

A third possibility is that this finding is related to our sample size. Because of the small number of participants who endorsed symptoms consistent with a diagnosis of BPD (*n* = 12), NPD (*n* = 10), ASPD (*n* = 2), or HPD (*n* = 6) exclusively (i.e., not comorbid with any other cluster B PD), we could not compare groups formed of specific disorders and decided to combine non-BPD cluster B PDs into a group. Nevertheless, a descriptive examination of the DBT skills use endorsed by those who reported symptoms consistent with only one of the cluster B disorders (see Table 3) showed that, at least numerically, the HPD or NPD groups seemed to have similar deficits in DBT skills use when compared to the BPD group. It is possible that with a larger sample and more stringent methodology, DBT skills deficits that are similar to BPD would also be found in NPD and HPD. Because we can only speculate on this result, additional research is needed to better characterize DBT skills deficits across PDs. Better understanding these differences in DBT skills deficits between cluster B PDs have important implications for treatment.

We also found that while both deficits in DBT skills use and emotion dysregulation were strong predictors of cluster B psychopathology, it appeared that the effect of emotion dysregulation accounted for the majority of the relationship between DBT skills use and severity of cluster B psychopathology. Thus, we can hypothesize that lack of DBT skills leads to emotion dysregulation, which in turn leads to more severe personality psychopathology. In other words, the effect of DBT skills use on cluster B psychopathology may be occurring through the regulation of emotions and, interestingly, fit the conditions under which the existence of a mediation model may occur [7]. This relationship needs additional investigation. Nevertheless, this finding strengthens the importance of teaching DBT skills to improve emotion regulation as a mechanism of change for cluster B psychopathology.

We further hypothesized that difficulties in emotion regulation and DBT skills use would be significant predictors of all four facets of psychopathy. This hypothesis was only partially supported. Deficits in DBT skills use predicted increased levels of callous affect and interpersonal manipulation, which is interesting considering these particular psychopathic characteristics are related to shallow emotions, lack of empathy and a high sense of self-worth [35]. Because these characteristics are related to the lack of emotional lability (and therefore skills to downregulate intense emotional experiences may not be beneficial) it is possible that some of the other DBT skills such as mindfulness or interpersonal effectiveness are lacking in those high on these traits. Although the DBT-WCCL does not permit us to examine specific DBT skills, mindfulness and interpersonal effectiveness could benefit those who have a disregard for others, are manipulative, superficial and lack remorse and empathy. Therefore, the current results lead us to hypothesize that teaching DBT skills may be a promising avenue for the treatment of interpersonal and affective deficits associated with psychopathy (although see [46] for possible contraindications).

We also found that participants who reported tendencies to be callous, to lack a sense of responsibility and have increased impulsivity, reported using more maladaptive strategies (e.g., blaming others, critical of self) for managing stress or solving problems in their lives. These results add to the existing body of literature highlighting the excess of maladaptive behaviors (e.g., drug use, promiscuity, aggression, suicidality, disregard for others) associated with psychopathy [19, 33, 53, 87]. Given that insufficient DBT skills use also predicted callous affect, it’s possible that teaching DBT skills may be a promising intervention to reduce maladaptive coping. However, problematic use of DBT skills did not predict an erratic lifestyle suggesting that DBT skills training interventions may not be best fitted to reduce maladaptive coping associated with this psychopathy dimension. While there is currently no research investigating the reduction of specific facets of psychopathy as a result of treatment, the current study’s findings suggest that DBT skills training may be appropriate for this population, although additional skills may be needed to address problems unique to psychopathy.

Neither facet of psychopathy was associated with problematic emotion regulation. These results add to a mixed body of literature connecting the impulsive facets of psychopathy to emotion dysregulation [20, 71]. For example, others have found that the erratic lifestyle and psychopathic criminal behaviors were correlated with a lack of understanding and ability to manage emotions [24], and self-centered impulsivity was also found to be associated to emotional dysregulation [20]. Our results contradict these findings, suggesting that the problematic behaviors associated with psychopathy may not be related to managing distress. Utilizing a community sample with lower overall psychopathy scores may have certainly influenced the findings. Therefore, different conceptualizations of psychopathy should be explored. Given the dynamic, heterogeneous nature of psychopathy [63] and inconsistent findings related to the investigation of effective treatments [67], more research to understand and characterize psychopathy is needed in order to improve psychotherapies.

There were several limitations in the current study. First, the analyses examining group differences were limited by a small sample size, which restricts the statistical power to detect significant differences. Our hypotheses should therefore be tested in larger samples. Second, questions can be raised concerning the personality disorders measure. In addition to the inherent issues regarding the use of self-report for diagnoses, the PDQ has suboptimal psychometric properties and therefore, the hypotheses need to be further analyzed with stricter measures where there is a lower likelihood of false positives. Also, the group names were maintained merely to denote PDs within the B cluster; the current study did not account for other non-cluster B PDs. In addition, the BPD group included comorbidity with any other cluster-B personality disorder. Therefore, it is difficult to differentiate the specific contribution of BPD on skills use (when compared to other cluster-B PDs). Follow-up studies with more rigorous methodologies need to be conducted before making too strong of inferences about these results particularly related to the diagnostic characteristics described in the current study. Third, one-third of the original sample was excluded due to responses deemed suspect on the PDQ validity scales. Considering psychopathy is associated with pathological lying, it is unclear if the suspect answers are a result of not paying attention to the questionnaire, or are an indicator of psychopathology. Fourth, the percentages of participants reporting symptoms consistent with cluster B PDs were higher than prevalence rates reported in previous epidemiological studies (e.g., [86]). While this discrepancy could be a result of the measure used, the authors also purposefully recruited for participants with the targeted difficulties; therefore the discrepancy between the norms and the current sample may also be due to oversampling. Fifth, the DBT-WCCL was developed on a sample of participants who met criteria for BPD but not other PDs. As a result, the interpretation of DBT-related skillful behavior outside of BPD may be biased. Future studies using the measure on various other populations is needed.

## Conclusion

Given the evolution of the diagnostic conceptualizations of PDs in the *DSM-5* and our understanding of transdiagnostic mechanisms across those disorders, future research exploring DBT skills use and emotion dysregulation in cluster B PDs and in psychopathy is warranted. Emotion regulation appears to have significant transdiagnostic relevance in the context of cluster B pathologies, but may have less relevance to psychopathy. Considering our findings that DBT skills use deficits may not be as pervasive as hypothesized, one way to interpret these results more broadly is that emotion regulation and DBT skills use may be much more nuanced than they are currently being conceptualized (or utilized). Taking a more multidimensional approach to measuring differences across pathologies is needed in both research and clinical practice to better understand core problems of behavior.

## Abbreviations

APA, American Psychological Association; ASPD, Antisocial Personality Disorder; AVPD, Avoidant Personality Disorder; BPD, Borderline Personality Disorder; CA, Callous Affect; CT, Criminal Tendencies; DBT, Dialectical Behavior Therapy; DBT-ST, Dialectical Behavior Therapy Skills Training; DBT-WCCL, Dialectical Behavior Therapy-Revised Ways of Coping Checklist; DERS, Difficulties in Emotion Regulation Scale; DSM-IV-TR, Diagnostic and Statistical Manual of Mental disorders, 4^th^ Edition (Text Revision); DSS, DBT Skills Use Subscale; ELS, Erratic Lifestyle; HPD, Histrionic Personality Disorder; IPM, Interpersonal Manipulation; NIMH, National Institute of Mental Health; NPD, Narcissistic Personality Disorder; PCL-R, Psychopathy Checklist-Revised; PD, Personality Disorder; PDQ-4+, Personality Disorder Questionnaire-4+; RDoC, Research Domain Criteria Project; SQ, Suspect Questionnaire Scale; SRP-SF, Self-Report Psychopathy Scale-Short Form; TG, Too Good Scale
